# Advanced User Interfaces for Neurorehabilitation

**DOI:** 10.1155/2014/740135

**Published:** 2014-07-10

**Authors:** Alessandro De Mauro, Belinda Lange, Andreas Dünser

**Affiliations:** ^1^eHealth and Biomedical Applications, Vicomtech-IK4, Paseo Mikeletegi 57, 20009 San Sebastian, Spain; ^2^Institute for Creative Technologies, University of Southern California, 12015 Waterfront Drive, Playa Vista, CA, USA; ^3^CSIRO, Computational Informatics, 15 College Road, Sandy Bay, Hobart, TAS 7005, Australia

A range of new user interfaces and systems have been developed as novel and relevant tools in neurorehabilitation, allowing the implementation and exploration of new rehabilitation approaches and protocols to be complemented and to improve upon traditional methods.

In this special issue both medical and engineering aspects of these tools as well as state-of-the-art research trends have been addressed in order to explore these types of solutions and how they can support and/or extend current clinical or home-based rehabilitation practices. The issue provides six original research articles by some of the leading experts in the field, covering development studies and studies investigating evidence of the effectiveness of new technologies, devices, specific applications, and treatment methodologies.

The paper of F. Trincado-Alonso et al. presents a study on the development of new strategies based on virtual reality (VR) that can provide additional information to clinicians regarding rehabilitation assessment. It includes the definition of set of metrics combining kinematic data in order to obtain parameters of reaching amplitude, joint amplitude, agility, accuracy, and repeatability during the evaluation sessions.

W. Chinthammit et al. report a pilot study that employs a prototype telerehabilitation system called “Ghostman”. It is a visual augmentation system designed to allow a physical therapist and patient to inhabit each other's viewpoint in an augmented real-world environment. The presented initial results look promising and suitable to be used in the field of stroke rehabilitation.

J. R. Octavia and K. Coninx focus on integrating adaptivity and personalization in rehabilitation training for multiple sclerosis patients. Their findings showed that adaptive personalized training trajectories have been successfully provided. Furthermore, they report on the development of positive social interaction during the collaborative rehabilitation training.

A. M. Fenuta and A. L. Hicks present a detailed comparison of two commercial rehabilitation treadmills in terms of oxygen demand and muscle activation during therapy. Important and clear indications on the use of both devices have been stated in the paper's conclusions.

C. Cortes et al. provide a mathematical formulation and implementation of a method to estimate the limb posture in VR and robotic assisted rehabilitation systems. Additionally, they present the details of a VR game platform for stroke rehabilitation and the quantitative assessment of the method during the analytic training of the elbow and wrist.

Finally, the paper of D. A. G. Galeano et al. proposes a novel balance training platform which combines postural analysis with synergistic electrical muscle stimulation and is based on low-cost gaming interfaces. Results include the technical validation of the platform using mediolateral and anteroposterior sways as basic balance training therapies.

As the editors of this special issue, we hope that readers will find these articles representative of the state-of-the-art in novel user interfaces for neurorehabilitation. This special issue highlights the latest research and developments in this important research field and the contributions it has made to improve treatment outcomes for patients, clinical research methodology, and the development of many practical applications.

